# Valence band offset of wurtzite InN/SrTiO_3 _heterojunction measured by x-ray photoelectron spectroscopy

**DOI:** 10.1186/1556-276X-6-193

**Published:** 2011-03-02

**Authors:** Zhiwei Li, Biao Zhang, Jun Wang, Jianming Liu, Xianglin Liu, Shaoyan Yang, Qinsheng Zhu, Zhanguo Wang

**Affiliations:** 1 Key Laboratory of Semiconductor Materials Science, Institute of Semiconductors, Chinese Academy of Sciences, P.O. Box 912, Beijing 100083, People's Republic of China

## Abstract

The valence band offset (VBO) of wurtzite indium nitride/strontium titanate (InN/SrTiO_3_) heterojunction has been directly measured by x-ray photoelectron spectroscopy. The VBO is determined to be 1.26 ± 0.23 eV and the conduction band offset is deduced to be 1.30 ± 0.23 eV, indicating the heterojunction has a type-I band alignment. The accurate determination of the valence and conduction band offsets paves a way to the applications of integrating InN with the functional oxide SrTiO_3_.

## Introduction

Group III nitrides have attracted much attention in recent years for their promising applications in high-power, high-speed devices [1,2]. Among the group III nitrides, indium nitride (InN), with a narrow direct band gap, small effective mass [3], and large electron saturation drift velocity [4], presents enormous potential for device applications such as near-infrared optoelectronics, high-efficiency solar cells, and high-speed electronics. Generally, InN is grown on foreign substrates such as sapphire, SiC, (111) silicon (Si) and GaAs. Strontium titanate (SrTiO_3 _or STO) single crystal with a cubic perovskite structure is also a good candidate. STO is often used to deposit functional oxide films which exhibit ferroelectricity, ferromagneticity, and superconductivity, so InN/STO heterojunction can integrate the superior optoelectronic properties of InN with the various functional characters of perovskites, and will be developed in the future. On the other hand, InN/STO heterojunction is a promising structure for fabricating optical and electrical devices since researchers found out that oxidation treatment can reduce the surface electron accumulation of InN film [5] (electron accumulation at the surface will prevent the realization of p-type conduction of InN). Furthermore, the integration of InN and STO may also be used to create a two-dimensional electron (hole) gas which leads to tailorable current-voltage characteristics [6]. In addition, STO has much larger dielectric constant than silicon dioxide (SiO_2_) and silicon nitride (SiN_x_), so it is an attractive candidate as an epitaxial gate oxide to replace SiO_2 _and SiN_x _for InN-based field effect transistor if band offset of InN/STO is partitioned approximately equally between valence and conduction-band edges. Although InN/STO shows many promising properties, there is a lack of experimental data on the interface band alignment parameters of the InN/STO heterojunction to date. X-ray photoeletron spectroscopy (XPS) has been demonstrated to be a direct and powerful tool for measuring the valence band offsets (VBOs) of heterojunctions [7-9]. In this paper, we present an experimental determination of the InN/STO VBO by XPS. Some problems related are also discussed to reveal the reliable results. Then the conduction band offset (CBO) is calculated by using the band gaps of the two materials.

## Experiment

Three samples were prepared in our experiment: a bulk commercial (111) STO substrate with the size of 10 × 5 × 0.5 mm^3^, a 300-nm-thick wurtzite (0001) InN layer grown on a (111) STO substrate and a wurtzite InN/STO heterojunction sample (a thin InN layer grown on a (111) STO substrate). The overlayer of the heterojunction sample formed the interface of interest must be sufficiently thin to allow XPS core levels from the underlying material to be probed due to the finite escape depth of the photoelectrons, its thickness was estimated to be 5 nm by the growth rate and growth time. Both of the two layers were grown by a horizontal-flow metal-organic chemical vapor deposition system at the temperature of 550°C. Before loading to the reactor, the (111) STO substrate was sequentially cleaned with organic solvents and rinsed with de-ionized water. InN films were grown on STO substrate with the trimethylindium and ammonia as the precursors and nitrogen as the carrier gas. Further details of the growth parameters are reported elsewhere [10]. XPS measurements were performed on a VG MKII XPS instrument with Al *Kα *(*hv *= 1486.6eV) as the x-ray radiation source, which had been calibrated on work function and Fermi energy level (*E*_F_). Because a large amount of electrons are excited and emitted from the samples, the samples are always positively charged. The electric field caused by the charge can affect the measured kinetic energy of photoelectrons, so all XPS spectra were calibrated by the C 1*s *peak (284.6 eV) from contamination to compensate the charge effect. Actually, the calibration to Fermi energy level was not necessary as it is the relative energy separation of spectral features that is of importance for the ultimate results. The surface of all samples were exposed to air, so the contaminations (e.g., oxygen and carbon) existing on the surfaces may affect the precise determination of the valence band maximum (VBM). To reduce the contamination effect, all the samples were subjected to surface clean procedure by argon positive (Ar^+^) bombardment with a voltage of 1 kV at a low sputtering rate, which alleviated damage to the samples. The reduced thickness was estimated to be 1 nm by the sputtering rate. After this process, the peaks related to contaminations were greatly reduced and no new peaks were introduced.

The VBO (Δ*E*_*V*_) is calculated from

(1) $$\Delta E_{V}=\Delta E_{\text{CL}}+[E_{\text{nl}}^{\text{STO}}-E_{\text{VBM}}^{\text{STO}}]-[E_{{\text{n}}^{\prime }{\text{l}}^{\prime \prime }}^{\text{InN}}-E_{\text{VBM}}^{\text{InN}}],$$

Where Δ*E*_*CL *_is the core-level (CL) separation between the n'1' core level of InN and the nl core level of STO, which is obtained from the InN/STO heterojunction sample. $\left[E_{\text{nl}}^{\text{STO}}-E_{\text{VBM}}^{\text{STO}}\right]$ and $\left[E_{{\text{n}}^{\prime }{\text{l}}^{\prime \prime }}^{\text{InN}}-E_{\text{VBM}}^{\text{InN}}\right]$ are the VBM energies with reference to core level peaks in STO and InN bulk constants obtained from the bulk STO sample and the 300-nm-thick InN layer, respectively. The VBM of each sample is determined by extrapolating a linear fit of the leading edge of the valence band photoemission to the baseline in order to account for broadening of the photoemission spectra [8,11,12].

## Results and discussion

**Figure 1:**
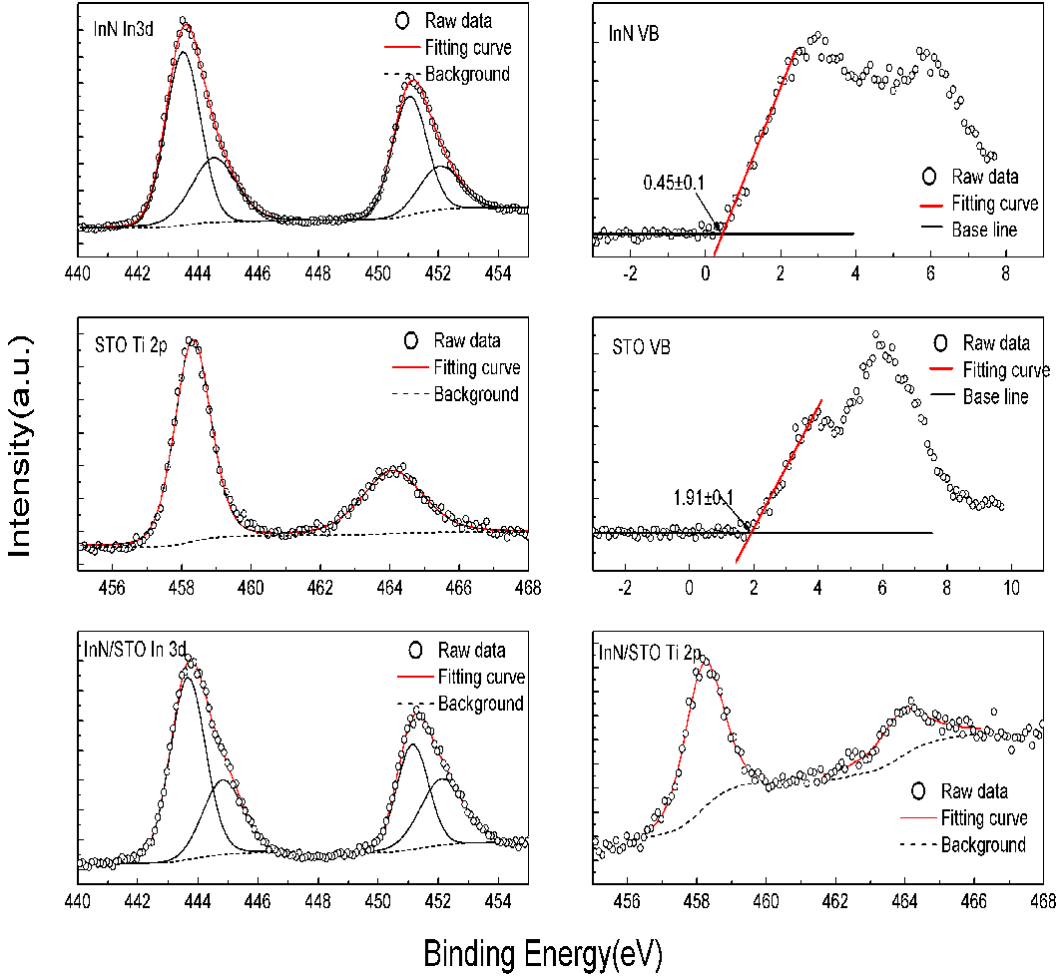
**Spectra of InN/STO sample**. 3*d *core level peaks for the InN and thin InN/SrTiO_3 _heterojunction samples, Ti 2*p *core level peaks for the SrTiO_3 _and InN/SrTiO_3 _heterojunction samples, and valence band photoemission for the InN and SrTiO_3 _samples. All peaks have been fitted using a Shirley background and Voigt (mixed Lorentzian-Gaussian) line shapes.

Figure 1 shows all the CL spectra including In 3*d *peak recorded on InN thick film and InN/STO samples, Ti 2*p *spectrum on bulk STO and InN/STO samples, as well as VB spectra recorded on InN and bulk STO samples. The CL spectra were fitted to Voigt (mixed Lorentzian-Gaussian) line shape by employing a Shirley background. The In 4*d *and Ti 3*p *semicore-level peaks used by other researchers [13,14] in similar experiments have not been chosen in the analysis as these levels are located at very low binding energy and hybridized with other shallow levels easily which will limit the accuracy of the results attained using these levels. Since considerable accordance of the fitted line to the original measured data has been obtained, the uncertainty of the CL position should be lower than 0.03 eV, as evaluated by numerous fitting with different parameters. The main uncertainty comes from the difficulty in determining the value of the VBM exactly. The peak parameters and the VBM positions are listed in Table 1 for clarity. In Figure 1 (InN), the In 3*d *spectrum include two peaks: 3*d*_5/2 _(443.50 eV) and 3*d*_3/2 _(451.09 eV) peaks, which are separated by the spin-orbit interaction with a splitting energy of 7.57 eV. With careful Voigt fitting, it was found out that both of the peaks consist of two components. The first In 3*d*_5/2 _component located at 443.50 eV is attributed to the In-N bonding [15], and the second, at 444.52 eV, is identified as being due to surface contamination. This two-peak profile of the In 3*d*_5/2 _spectra in InN is so typical and have been demonstrated by other researchers [16-20]. Comparing their binding energy separation with previous results [19,21,22], we suggest to assign the second peak at 444.52 eV to the In-O bonding which is due to contamination by oxygen during the growth process. The ratio of In-N peak intensity to the oxygen-related peak indicates that only a small quantity of oxygen contamination exists in our samples. The Ti 2*p *spectrum (STO in Figure 1) also consists of two components: 2*p*_3/2 _(458.19 eV) and 2*p*_1/2 _(464.09 eV) peaks. Both of them are quite symmetric indicating the uniform bonding state and good quality of our sample. Using the linear extrapolation method mentioned above, the VBM of InN and STO are 0.45 ± 0.1 eV and 1.91 ± 0.1 eV, respectively. The spectra of InN/STO sample are shown in Figure 1 (InN/STO). Compared with the spectra recorded on the InN and STO samples, the In 3*d *core level is shifted to 443.68 eV and Ti 2*p *is shifted to 458.17 eV. The VBO value is calculated to be 1.26 ± 0.23 eV by substituting those values into Eq. (1).

**Table 1:** XPS core level fitting results and VBM positions

Sample	State	Binding energy(eV)
InN	In 3*d*_5/2_	443.50 ± 0.03
		
		444.52 ± 0.03
	
	VBM	0.45 ± 0.1

SrTiO_3_	Ti 2*p*	458.19 ± 0.03
	
	VBM	1.91 ± 0.03

InN/SrTiO_3_	In 3*d*_5/2_	443.68 ± 0.03
		
		444.87 ± 0.03
	
	Ti 2*p*	458.17 ± 0.03

VBMs are obtained by linear extrapolation of the leading edge to the extended base line of the VB spectra.

Reliability of the analysis of the measured results is provided by considering possible factors that could impact the experimental results. InN is a kind of piezoelectric crystal, so the strain existing in the InN overlayer of the heterojunction will induce piezoelectric field and affect the results. According to the previous reports, we know the lattice mismatch between InN and STO is larger than 9.8% inferred from the Φ scanning patterns of InN film grown on STO [10].The majority of the strain relaxes within the first few monolayers in the InN film, so the InN layer can be approximately treated as completely relaxed and this approximation should not introduce much error in our result. In addition, InN always exhibits obvious electron accumulation at its surface and causes the band bending downward (approximately 0.6 eV) near the surface [23-25]. Theoretical calculations revealed that the electron accumulation thickness was estimated to be approximately 5 nm [23-25]. The band bending could also impact the measured VBO values of heterojunction [24]. However, the thin InN/STO of heterojunction sample is only 4 nm after the cleaning process, so the thin overlayer can be treated as consisting of surface and interface, and the band-bending effect can be neglected in this experiment. Since the factors that can affect the results can be excluded from the measured results, the experimental obtained VBO value is reliable.

**Figure 2:**
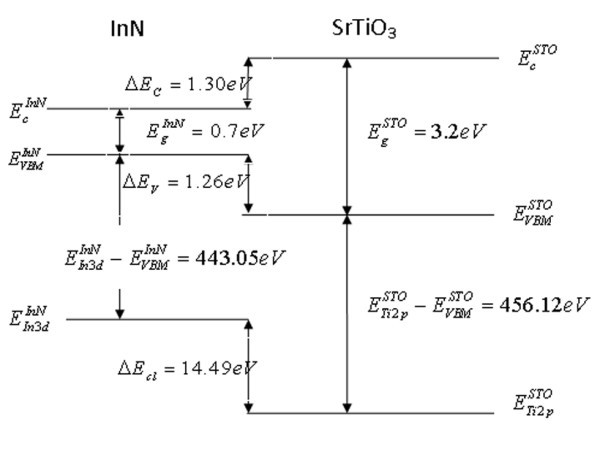
**Schematic representation of the band line-up at an InN/SrTiO**_**3 **_**heterojunction at the room temperature**. A type-I heterojunction is formed in the straddling configuration.

Making use of the band gap of InN (0.64 eV) [26] and SrTiO_3 _(3.2 eV) [27], the CBO (Δ*E*_*C*_) is calculated to be 1.30 eV and the ratio of Δ*E*_*C*_/Δ*E*_*V *_is close to 1:1. As shown in Figure 2, a type-I heterojunction is seen to be formed in the straddling configuration. As mentioned above, STO can be utilized as the gate oxide for InN-based metal-oxide semiconductor and the gate leakage is expected to be negligible because of the large CBO between STO and InN, which is different from the Si-based devices [28].

## Summary

In conclusion, we have measured the VBO of an InN/SrTiO_3 _heterojunction by XPS. All the samples were carefully cleaned by Ar^+ ^bombardment before the measurement, and the intensity of contamination elements peaks is greatly reduced. The measured VBO is 1.26 ± 0.23 eV. The main factors that may impact the measured result are discussed. The CBO is deduced to be 1.30 ± 0.23 eV. This offset causes a type-I heterojunction between InN and SrTiO_3 _in the straddling arrangement and proves that STO can be utilized as the gate oxide for InN-based metal-oxide-semiconductors devices.

## Competing interests

The authors declare that they have no competing interests.

## Authors' contributions

ZL carried out the experiments and wrote the original paper. BZ, JW and JL prepared the samples and analyzed the results. XLL, SYY and QSZ participated in the design of the study and collected the references. ZGW helped to revise the original manuscript. All authors read and approved the final manuscript.
